# Secretions of mandibular glands are not involved in the elicitation of rescue behaviour in *Formica cinerea* ants

**DOI:** 10.1007/s00040-017-0547-x

**Published:** 2017-02-09

**Authors:** K. Miler, K. Kuszewska

**Affiliations:** Institute of Environmental Sciences, Jagiellonian University, Gronostajowa 7, 30-387 Kraków, Poland

**Keywords:** Mandibular gland, Pheromone signalling, Reduced communication, Rescue behaviour

## Abstract

Certain ants perform rescue behaviour for other ants that require help, and the expression of rescue behaviour is hypothesized to depend on signals (“calls for help”) sent by the imperilled individuals. We studied whether the mandibular glands were involved in the elicitation of rescue behaviour in *Formica cinerea* Mayr ants. In the first experiment, we determined the occurrence and characteristics of rescue behaviour directed towards nest mates with impaired mandibular gland communication. We did not observe any difference in rescue behaviour directed towards individuals who were untreated, treated with paint over the mandibles, or sham-treated with paint over the thorax. In the second experiment, we determined whether rescue behaviour would occur towards dummy ants coated with the contents of the mandibular glands. Compared with the control untreated nest mates, we found that rescue behaviour was not directed towards either the untreated dummy ants or the dummy ants covered with crushed mandibular glands. Our results indicated that the “call for help” signal does not originate from the mandibular glands. Therefore, we propose that gaster-tip glands represent a plausible alternative source of rescue-eliciting pheromone(s) for the *F. cinerea* ants examined in this study.

**Electronic supplementary material:**

The online version of this article (doi:10.1007/s00040-017-0547-x) contains supplementary material, which is available to authorized users.

Chemical communication among nest mates plays a crucial role in the functioning of an ant colony. For example, the group predation of ponerine ants occurs via recruitment behaviour based on pheromones secreted by scouting individuals (Maschwitz and Schönegge 1977), and the queens of Pharaoh ants produce pheromones that enable their recognition by workers and special functions in the colony (Edwards and Chambers 1984). Pheromones in ants are likely involved in every aspect of their lives and ensure colony integrity (Jackson and Morgan 1993). One of the best examples of the complex social behaviour controlled by pheromones is provided by certain sand-dwelling ants, including the species in this study, *Formica cinerea* Mayr, showing rescue behaviour towards nest mates that require help (Czechowski et al. 2002). Indeed, the elicitation of rescue behaviour, specifically in *Formica*, is hypothesized to depend primarily on pheromonal signals (“calls for help”) sent by the imperilled individuals (Czechowski et al. 2002). Thus, we studied whether mandibular glands were involved in the expression of rescue behaviour in *F. cinerea* ants. These glands are the most likely candidates for the source of rescue-eliciting pheromone(s) because of their involvement in the related functions, e.g., coordinating, alerting, and attracting (Attygalle and Morgan 1984; Ali and Morgan 1990). To demonstrate the potential importance of secretions from mandibular glands, we designed two experiments in which mandible-based pheromone communication was blocked between nest mates or the contents of the mandibular glands was used to provoke the expression of rescue behaviour. Our methods were similar to those used in previous studies (e.g., Hölldobler et al. 2013; Stuttard et al. 2016).

In the first experiment, the ants were tested in dyadic encounters of individuals from the same colonies. In each test, one ant required help, as it was entrapped on the surface of the sand (namely, the entrapment bioassay, e.g., Nowbahari et al. 2009; 2012), whereas the nest mate was free. The entrapped ant was either untreated (control group), had a drop of paint applied over the mandibles (group with blocked pheromone communication via mandibular glands), or had a drop of paint applied over the thorax (sham-treated group). In each test, we noted whether the free ant performed rescue behaviour, the latency to the first episode of rescue, and the total duration of rescue. Digging around the entrapped nest mate, pulling at its limbs, transporting sand particles away from it, and biting the snare entrapping the nest mate were evaluated as the main subcategories of rescue behaviour. In the second experiment, the ants were tested in an analogous situation, but the ‘trapped’ ant was either untreated (first control group) or a dummy ant that was either untreated (second control group) or covered in the crushed contents of a mandibular gland (experimental group). The same type of data was collected in the second experiment, and the same subcategories of rescue behaviour were evaluated. We used a two-tailed Fisher’s exact test (FET) to detect the between-group differences in the rate of occurrence of rescue behaviour and a Kruskal–Wallis ANOVA to detect the between-group differences in the latency and the duration of the behaviours (see the Supplementary Information for detailed descriptions of the materials and methods).

In the first experiment, we found that rescue behaviour occurred in 16 of 30 tests with the first untreated control group of ants, in 12 of 30 tests with the second mandible-treated group of ants, and in 11 of 30 tests with the third thorax-treated group of ants. Based on these results, the frequency of rescue behaviour occurrence among the groups was not significantly different (FET yielded nonsignificant results for each comparison). In addition, differences were not observed among these three groups in either the latency to the first episode of rescue (K–W ANOVA: *H* = 0.123, *p* = 0.940) or the total duration of rescue (K–W ANOVA: *H* = 0.192, *p* = 0.908). In the second experiment, we found that rescue behaviour occurred in 31 of 60 tests with the first untreated control group of ants, in none of the 60 tests with the third control group of dummy ants, and in 4 of 60 tests with the second gland-treated group of dummy ants. These results indicated that only the live ants in the first group elicited rescue behaviour (FET yielded a nonsignificant result for the comparison between the second and the third groups).

The data from the first experiment could be confounded by certain minute residual pheromone(s) on the body surface of trapped ants with blocked pheromone communication via the mandibular glands [i.e., these ants could discharge rescue-eliciting pheromone(s) originating from the mandibular glands before the experimental procedure, which would explain the subsequent rescue behaviour]. However, the effects of pheromone residuals were unlikely, because in the second experiment, the contents of the mandibular glands did not elicit rescue behaviour towards the dummy ants. All four attempted rescues of the gland-treated group of dummy ants were weak and could have resulted from other substances transferred onto them during experimental procedures (Bagnères et al. 1991). Thus, our results indicated that the mandibular glands are not involved in the elicitation of rescue behaviour in *F. cinerea* ants.

The previous reports have indicated that the mandibular gland secretions of ants function at a minimum to attract conspecifics (Cammaerts et al. 1981; Howard et al. 1982) and release both alarm and digging behaviours, which are responses involved in rescue operations (Wilson 1958; McGurk et al. 1966). However, these studies involved ants from genera that were not used in the present study (*Formica*), including *Pogonomyrmex, Wasmannia*, and *Myrmica*. In addition, the mandibular glands in *Formica* workers contain low quantities of volatile materials (Bagnères et al. 1991). Therefore, other glands in *Formica* are most likely involved in rescue elicitation, such as Dufour glands, which function in communication (Löfqvist 1976; Attygalle and Morgan 1984). An alternative or complementary explanation could be that the production of CO_2_ by nest mates that require help attracts other ants and releases the basic forms of rescue behaviour, alarm, and digging behaviours, as observed in *Solenopsis* ants (Hangartner 1969). Notably, stridulation may be an alternative mode of communicating for help. Indeed, stridulation is hypothesized to have evolved among the ants to alert nest mates that rescue is required, although this hypothesis has been largely rejected (Golden and Hill 2016). Moreover, stridulation as a call for help is not relevant in *Formica*, because stridulatory organs are absent in this genus (Czechowski et al. 2002). Thus, the “call for help” in our study species of *Formica* could not have involved vibroacoustic signals.

The current studies on the selected ecological and evolutionary aspects of rescue behaviour are strongly dependent on the hypothesis that individuals who require help emit “call for help” signals; however, this behaviour remains largely unknown (e.g., Nowbahari et al. 2009; Miler 2016), and uncovering the mechanism of rescue behaviours in ants is an essential component of further research. Although mandibular gland secretions did not elicit rescue behaviours in this study, they can possess such a function in other sand-dwelling ants which display rescue behaviours (Hollis and Nowbahari 2013). In *F. cinerea* ants as well as other species of this genus that display rescue behaviours, however, “gaster-tip” gland secretions should be investigated in further studies on the glandular origins of the rescue-eliciting pheromone(s).

## Electronic supplementary material

Below is the link to the electronic supplementary material.

Supplementary material 1 (DOC 46 KB)
