# Supplementary material for: Secretions of mandibular glands are not involved in the elicitation of rescue behaviour in Formica cinerea ants
Source: Insectes Soc. 2017 Feb 9;64(2):303–5. doi: 10.1007/s00040-017-0547-x (PMC5397451; doi:10.1007/s00040-017-0547-x)
Supplement: Supplementary file 1 — Supplementary material 1 (DOC 46 KB) [file 40_2017_547_MOESM1_ESM.doc]

Secretions of mandibular glands are not involved in the elicitation of rescue behaviour in *Formica cinerea* ants

Krzysztof Miler and Karolina Kuszewska

Correspondence: Krzysztof Miler; E-mail: krzysztof.miler@uj.edu.pl

Supplementary Information

Two experiments were performed. In the first experiment, active *Formica cinerea* Mayr foragers from three different colonies were collected from the Błędowska Desert near Olkusz in southern Poland. Ants from each colony were kept in separate open plastic boxes (25 x 17 x 10 cm), and the walls were covered in escape-preventing Fluon® (oobleck, Poland) at a constant ambient temperature of 24°C and relative humidity of 40-60%. The ants were provided with water and honey *ad libitum* and allowed to acclimatize in these conditions for 48 hours after transportation from the field, and then the tests were performed. We used the entrapment bioassay in the form of dyadic encounters between an entrapped ant and a free nest mate (potential rescuer). In each entrapment test, a plastic cup (7 cm diameter, 10 cm height) was filled with dry sand. The entrapped ants were tied by the petiole to a small 1.5 cm diameter piece of filter paper (Munktell, Germany) using nylon thread (insel, Poland) and placed inside the cup on the surface of the sand; however, the filter paper was not visible, and the ant was only slightly covered with sand particles. Immediately after the entrapped ant was positioned, a potential rescuer was introduced into the cup. Each test began with the introduction of the potential rescuer and continued for 5 minutes. Three types of entrapped ants were used: (1) untreated control ants, (2) ants with a drop of paint applied at the base of the mandibles (group with blocked pheromone communication via the mandibular glands), and (3) ants with a drop of paint applied over the thorax (sham-treated group). We used a white, quick-dry paint marker (Sakura, Japan) as the source of the paint, applied by means of insect pins. The paint was allowed to dry for a few seconds while the treated ant was held in forceps, and then the ant was tied to the filter paper and placed inside the test cup. We performed 10 tests for each group for each colony (90 tests in total). In each test, we noted whether rescue behaviour occurred and when such behaviour was observed, and we recorded the latency to the first episode of rescue behaviour and the total duration of rescue behaviour. Digging around the entrapped nest mate, pulling at its limbs, transporting sand particles away from the entrapped ant, and biting of the snare entrapping the nest mate were evaluated as the main subcategories of rescue behaviour. In the second experiment, active foragers of the same species from three other colonies were collected again from the same site and kept in the abovementioned laboratory conditions. The ants were acclimated for 24 hours, and then the tests were performed. An analogous entrapment bioassay to that of the first experiment was then performed. The entrapped individual included (1) an untreated control ant, (2) an untreated dummy ant, or (3) a dummy ant covered with the crushed mandibular gland of a nest mate of the potential rescuer. The dummy ants were 1 cm long pieces of wooden toothpicks. The mandibular glands were obtained by decapitating the ant, dissecting the head and then, using forceps, extracting the left mandible and mandibular gland. In the second group, the “entrapped dummy ants” were placed in test cups and then each was smeared with the mandibular gland, which was crushed in the process. Dissections were performed in sterile conditions, and the tools (microscissors, forceps, insect pins, etc.) were sterilised after each dissection in 98% ethanol. We performed 20 tests per group for each colony (180 tests in total). Similar to the first experiment, the same type of data was collected with the same subcategories of rescue behaviour.

We used a two-tailed Fisher’s Exact Test (FET) to detect the between-group differences in the rate of rescue behaviour occurrence and a Kruskal-Wallis ANOVA to detect the between-group differences in the latency and duration of the behaviours. Statistical analyses were performed using the STATISTICA 12.5 software (StatSoft, Poland).
